# How is GPS used? Understanding navigation system use and its relation to spatial ability

**DOI:** 10.1186/s41235-024-00545-x

**Published:** 2024-03-19

**Authors:** Alexis Topete, Chuanxiuyue He, John Protzko, Jonathan Schooler, Mary Hegarty

**Affiliations:** 1 Department of Psychological and Brain Sciences, University of California, Santa Barbara, USA; 2 Rutgers Center for Cognitive Science, Rutgers University, New Brunswick, USA; 3 Department of Psychological Science, Central Connecticut State University, New Britain, USA

**Keywords:** Spatial ability, Navigation, GPS use, GPS dependence

## Abstract

Given how commonly GPS is now used in everyday navigation, it is surprising how little research has been dedicated to investigating variations in its use and how such variations may relate to navigation ability. The present study investigated general GPS dependence, how people report using GPS in various navigational scenarios, and the relationship between these measures and spatial abilities (assessed by self-report measures and the ability to learn the layout of a novel environment). GPS dependence is an individual’s perceived *need* to use GPS in navigation, and GPS usage is the *frequency* with which they report using different functions of GPS. The study also assessed whether people modulate reported use of GPS as a function of their familiarity with the location in which they are navigating. In 249 participants over two preregistered studies, reported GPS dependence was negatively correlated with objective navigation performance and self-reported sense of direction, and positively correlated with spatial anxiety. Greater reported use of GPS for turn-by-turn directions was associated with a poorer sense of direction and higher spatial anxiety. People reported using GPS most frequently for time and traffic estimation, regardless of ability. Finally, people reported using GPS less, regardless of ability, when they were more familiar with an environment. Collectively these findings suggest that people moderate their use of GPS, depending on their knowledge, ability, and confidence in their own abilities, and often report using GPS to augment rather than replace spatial environmental knowledge.

**Supplementary Information:**

The online version contains supplementary material available at 10.1186/s41235-024-00545-x.

## Significance statement

With the exponential rate at which technology is advancing in usability and accessibility to the general public, one important issue is the association between using these technologies and cognitive ability. The use of GPS navigation aids, especially on mobile phones is a case in point, with little research on how using this technology is associated with our ability to navigate on our own, without an aid. There is even less research on *how* people report using GPS and how use is associated with both perceptions of navigation ability (measured by self-reports) and objective navigation ability (e.g., as measured by ability to learn the layout of a novel environment). This paper provides new information on how navigation ability is related to reported dependence on GPS in general and for specific uses; turn-by-turn directions, route planning (i.e., looking at an overview of a route before actually navigating), time and traffic estimates, and finding a specific service (e.g., a gas station) along a route. It suggests that while less able navigators report being more dependent on GPS and using it more for turn-by-turn directions, people vary their use of GPS, depending on their familiarity with an environment and most often report using functions such as time-and-traffic estimates that provide information on current conditions, rather than replacing enduring knowledge of spatial layout.

The average person has daily access to a device, a smartphone, with substantially faster processing power than the original technology that was used to land Neil Armstrong on the moon. Smartphones have allowed people to connect with people around the world at any time, access breaking news as it happens, and instantly know the most recommended places to visit when traveling. However, there is an increasing concern that a particular function of the smartphone—navigation systems—may have a detrimental impact on the acquisition and maintenance of both spatial knowledge of the environment and actual navigation ability (Dahmani & Bohbot, 2020; Ishikawa, 2019; McKinlay, 2016; Ruginski et al., 2019). Digital navigation devices rely on Global Navigation Satellite Systems (GNSS) and are commonly referred to as global positioning system (GPS) navigation systems, so we will hereafter refer to them as GPS navigation systems, or simply GPS.

This concern is not unfounded—an early study found that when asked to navigate with GPS navigation devices rather than traditional navigation aids, such as paper maps or verbal instructions, people performed more poorly on measures of spatial knowledge acquisition (drawing sketch maps and pointing to landmarks learned), and also reported greater wayfinding difficulty (Ishikawa et al., 2008). Similarly, people who learned the layout of an environment via a mobile map performed worse on a distance estimation task than those who learned via a paper map (Willis et al., 2009). Moreover, in a study of route learning and scene recognition in a simulated driving task, audible turn-by-turn directions (similar to GPS verbal directions) impaired scene recognition and resulted in longer travel time when attempting to replicate a learned route (Fenech et al., 2010).

Experimental studies in virtual environments have also examined GPS detriments on navigation ability. In an eye tracking study, participants who spent more time looking at a GPS-like map when traveling through a virtual environment took longer wayfinding paths without GPS, acquired less spatial knowledge, and reported a poorer sense of direction (Hejtmánek et al., 2018). In other studies, using a GPS-like aid when navigating a virtual environment impaired spatial memory as measured by landmark recall, map reconstruction, and pointing to unseen landmarks (Gardony et al., 2013, 2015).

A number of theoretical explanations have been proposed for the negative effects of GPS on acquisition of spatial knowledge. Willis et al (2009) proposed that mobile map users acquire more fragmented knowledge of space, whereas physical map users acquire a more globally consistent representation of their environment. An alternative theory (proposed by Fenech et al., 2010) is that the turn-by-turn direction function of GPS may cause inattention blindness, a perceptual failure to attend to (or “see”) elements in an environment (Simon & Chabris, 1999; Mack, 2003) and this contributes to poor wayfinding and scene recognition when navigating. A related explanation is that GPS impairs navigation and spatial memory due to divided attention—that is, that having to switch between attending to the GPS device and the environment (Gardony et al., 2013, 2015; Hejtmánek et al., 2018).

## Relations between GPS use and navigation ability

A number of recent studies have found that reported GPS use is related to individual differences in navigation and other spatial abilities (Dahmani & Bohbot, 2020; He & Hegarty, 2020; Ishikawa, 2019; Miola et al., 2023; Ruginski et al., 2019). Specifically, more dependence on GPS has been associated with a poorer self-reported sense of direction and more spatial anxiety (He & Hegarty, 2020; Hejtmánek et al., 2018; Miola et al., 2023), poorer performance on mental rotation and perspective taking abilities (Ruginski et al., 2019), and less ability to learn the layout of new places (Ishikawa, 2019; Ruginski et al., 2019). For example, more frequent use of GPS is related to performance in the Virtual SILCton task (Weisberg et al., 2014) which measures ability to learn the layout of new environments, and this relation is mediated by direct negative effects of GPS use on mental rotation and perspective taking (Ruginski et al., 2019). Self-reported GPS dependency has also been found to be positively associated with higher levels of spatial anxiety and a poorer self-reported sense of direction, as well a fixed mindset regarding navigation ability and a lower tendency to explore one’s environment (He & Hegarty, 2020; Miola et al., 2023).

In general, two accounts of these results have been proposed. First, it is possible that people who depend on GPS for navigational assistance over time do not exercise their own navigation ability, and consequently lose ability to navigate on their own (i.e., navigation ability is a “use it or lose it” cognitive skill, McKinlay, 2016). Another possibility is that people with an existing poor sense of direction and greater spatial anxiety use GPS more frequently because they are not confident in their ability to navigate on their own. These two explanations are not mutually exclusive and might work in tandem such that people who are not confident in their navigation abilities rely on using GPS to navigate in environments, consequently exercising their navigation abilities less, and so on.

A couple of recent studies have examined effects of long-term reliance on GPS and navigation ability. Ishikawa (2019) found that greater accumulated use of in-car navigation systems over time resulted in poorer wayfinding efficiency and poorer memory for route configurations, and that these effects were independent of self-reported sense of direction and spatial aptitudes, such as mental rotation. Dahmani and Bohbot (2020) also found relations between habitual use of GPS and spatial memory. In a longitudinal study, they tested participants’ navigation abilities in a laboratory task (a radial arm maze task) and then following up with them 3 years later, at which time they reported their habitual GPS use and were retested. Those who reported more GPS use in the intervening years used fewer spatial memory strategies (e.g., using distal cues to remember where things are) and more response-based strategies (e.g., counting out the number of arms, or remembering whether they go left or right at a specific landmark) than they did in the initial phase (Dahmani & Bohbot, 2020). This effect persisted even when people did not change their perceived, self-reported navigation abilities. The researchers concluded that extensive GPS use caused a detriment in their navigation ability. While a promising result, longitudinal testing was not part of the original design in this study, so the sample was small (only 13 participants).

In studies to date, researchers have asked about GPS dependency and use in a very general sense. However, people use GPS navigation devices in different ways (e.g., for time and traffic estimates, route planning before a trip, and searching for specific services along a route). These can be used in tandem with turn-by-turn navigation, or in isolation. Additionally, turn-by-turn directions can be used more or less, from the entire route being traveled, to a subsection of the route (e.g., for the last few turns, or to know the name of a street or exit). Some functions of GPS, such as time and traffic estimates or route planning before a trip, are assumed to supplement acquired spatial knowledge. For example, having an excellent cognitive map of an environment does not provide information on dynamic situations such as a recent crash on the highway that will impact travel time. Additionally, the route planning function is more analogous to learning via a map prior to travel, which has been shown to be a contributor to developing survey knowledge of an environment (Thorndyke & Hayes-Roth, 1982).

Understanding how people of different navigation abilities use GPS represents a current gap in our knowledge. As a result, we do not know whether more use of GPS in general is associated with a poor sense of direction, or if it is overuse of specific GPS functions that accounts for the negative relations between GPS and navigation ability observed in previous literature. Additionally, it is unclear whether people modulate use of their GPS in accordance with their familiarity with their current navigation goal. For example, more adaptive use might include utilizing GPS within novel environments, but not in navigation scenarios familiar to the user. In sum, it is important to study when people use GPS to augment their internal representations of environments, as opposed to using it as an external representation that replaces the need to construct internal spatial representations.

To date, few studies have looked at effects of GPS use on objective (as opposed to self-report) spatial measures, and some of those have significant limitations. For example, Muffato et al. (2022) found that GPS use was negatively associated with visuospatial working memory, but did not include an objective measure of navigation. Ruginski et al. (2019) used a robust objective navigation measure (the SILCton task, Weisberg et al., 2014), but their measure of GPS use was limited to one item. Dahmani and Bohbot (2020) also had robust objective measures for navigation, but the final sample size for their longitudinal design was small (13 participants). Finally, Ishikawa (2019) used a thorough self-reported assessment of GPS reliance and accumulated use, but the navigation measure in this study only contained three trials, limiting its reliability. Thus, there is a need to investigate whether GPS affects objective navigation ability in a well-powered study with robust measures of GPS dependence and use and navigation ability.

## The present study

The purpose of the study is to examine if, and how, self-reported use of various GPS functions correlate with self-reported navigation ability, spatial anxiety, and navigation performance (as measured by ability to learn the layout of a novel environment). In two preregistered studies (see https://osf.io/yn4jr/ and https://osf.io/bzcrp/), one online and one in-person, we examined how reported use of different GPS functions varies across individuals and situations, and how this variation is related to measures of navigation ability. Specifically, we addressed the following questions:

1. How are self-reports of navigation ability (sense of direction) and spatial anxiety related to GPS use and dependency?
2. How do people use GPS and moderate their use of GPS based on familiarity of the navigation scenario?
3. How is objectively measured navigation ability related to GPS dependency and use?

To answer these questions, we introduced a new questionnaire to measure the following ways in which people report using GPS for: (1) turn-by-turn directions (using GPS to give real-time directions to a destination), (2) route planning (using GPS to look at the overall route to a destination prior to actually navigating), (3) time and traffic estimates (using GPS to assess estimated time of arrival and potential traffic that may affect route choice and travel time), and 4) finding a specific service (using GPS to find particular stops on the way to a destination, such as a gas station, coffee shop, or restaurant). We also measured general dependence on GPS, that is, an individual’s perceived *need* to use GPS in navigation, using an existing questionnaire (He & Hegarty, 2020). In two studies, we examined how GPS uses and dependency related to navigation ability and anxiety. The first study was conducted online and used self-report measures of navigation ability and anxiety. The second study was in-person and added an objective measure of navigation ability; the Virtual SILCton task (Weisberg et al., 2014).

We predicted that greater reported use of GPS for turn-by-turn directions would be associated with a poorer self-reported sense of direction (SOD) and positively associated with overall GPS dependence. This prediction is consistent with both the possibility that people who are less confident in their own sense of direction abilities, rely more on a navigation aid and also with the possibility that greater use of GPS leads to the decline of navigation abilities over time (e.g., Dahmani & Bohbot, 2020; He & Hegarty, 2020; Ishikawa, 2019; Ruginski et al., 2019). Inversely, we predicted that more reported use of the route planning function would be positively associated with SOD, as this function is most similar to reading a map or atlas, which is associated with a good SOD and assists spatial knowledge acquisition (e.g., Ishikawa et al., 2008; Willis et al., 2009*).* We expected no significant association between SOD and reported time and traffic estimate use or finding a specific service, as these functions are assumed as being supplemental to spatial knowledge of an environment. We also attempted to replicate the findings of He & Hegarty who found that GPS dependency was related to greater spatial anxiety, less tendency to explore one’s environment, and less growth mindset with respect to navigation.

With regard to familiarity, it was expected that people would report using each GPS function less in more familiar environments (i.e., people would moderate their GPS use with familiarity). However, we expected that those with low reported spatial ability would report less moderation of their GPS use with familiarity, based on previous evidence that they are more dependent on GPS in general (He & Hegarty, 2020). We also analyzed how frequently people report to using each function of GPS in general, and across navigation scenarios.

Finally, we predicted that people who reported greater general dependency on GPS and more use of GPS for turn-by-turn directions would have poorer objective navigation performance, as measured by Virtual SILCton (Weisberg et al., 2014). Virtual SILCton is based on real-world navigation studies (Ishikawa & Montello, 2006; Schinazi et al., 2013), and employs a route-integration paradigm to measure spatial knowledge acquisition (i.e., people follow a series of guided routes, and are tasked with remembering the locations of various landmarks in the environment). This measure was chosen as some of the most influential studies of navigation ability to date have focused on the ability to learn the layout of a novel environment (e.g., Ishikawa & Montello, 2006; Hegarty et al., 2006; Weisberg et al., 2014; Weisberg & Newcombe, 2016; Youngson et al., 2019), and it was the measure used by Ruginski et al. (2019) in their pioneering study of GPS use and objectively measured navigation ability. We also included measures of mental rotation and spatial perspective taking in our studies, to attempt to replicate Ruginski’s mediation results.

## Method (online study)

### Participants

A total of 200 participants were recruited through the UC Santa Barbara Psychology Research participant pool to participate in an online study. This sample size was chosen based on an a priori power analysis which revealed that *N* = 191 is the minimum sample size necessary to achieve 80% power for detecting a small to medium effect (*r* = 0.20), at a significance criterion of Alpha = 0.05. In the study, 12 participants were removed from analyses due to indicating that they either did not take the study seriously, did not consent, or did not complete the study, for a total of 188 participants included in analyses. Participants ranged from 18 to 29 years old (*M* = 19.54, SD = 1.47, 135 females). Most of the participants were White (*n* = 85, 45.2%) or Asian (*n* = 48, 25.5%), with the remaining participants identifying as Multiracial (*n* = 16, 8.5%), Black or African American (*n* = 3, 1.6%), Native Hawaiian or Pacific Islander (*n* = 2, 1.1%), American Indian or Alaska Native (*n* = 1, < 1%), or other (*n* = 33, 17.6%).

### Materials

Participants were administered all measures via a survey on the Qualtrics online platform. The following variables were included.

#### Measure of GPS dependence

The GPS Dependency Scale (He & Hegarty, 2020) assesses how much a participant reports depending on using GPS in 8 different navigation scenarios on a 5-point rating scale, 1 = Never, 5 = Always. Responses are averaged across questions, with a higher score indicating greater reliance/dependence on using GPS in navigation (see Additional file 1: Table S1 for individual items and their descriptive statistics).

#### Measure of GPS use

The GPS Usage scale was developed from a preliminary study of 30 undergraduate students, who provided open-ended responses reporting how they used GPS in different navigation scenarios (e.g., traveling to a brand-new place, somewhere that is semi-familiar, somewhere very familiar, etc.). From these responses, 4 most common functions were identified (turn-by-turn directions, route planning, time and traffic estimates, and finding a specific service). The questionnaire described 8 different navigation scenarios and asked participants to indicate how often they use the 4 different GPS functions for each scenario (see Appendix^1^). Participants responded on a 3-point scale, never (coded as 0), sometimes (coded as 1), and often/always (coded as 2). Individual items and their descriptive statistics are listed in Additional file 1: Table S2.

#### Measure of self-reported sense of direction

The Santa Barbara Sense of Direction (SBSOD) scale (Hegarty et al., 2002) assessed self-reported navigation ability. This scale consists of 15 questions, with 7 stated negatively (e.g., I don’t have a very good “mental map” of my environment), and 8 stated positively (e.g., I can usually remember a new route after I have only traveled it once). Negatively-worded items were reverse-scored, and the average of the 15 items was the measure participants’ perceived sense of direction, on a 7-point scale. A higher score indicates a better perceived sense of direction (SOD).

#### Measure of spatial anxiety

The Spatial Anxiety scale (Lawton, 1994), as modified by He and Hegarty (2020), assessed self-reported level of anxiety in different navigation scenarios. This scale contains 13 items, and is on a 5-point scale. Responses are averaged across items and a higher score indicates greater spatial anxiety.

#### Measure of exploration tendency

The Exploration Tendency scale (He & Hegarty, 2020), was administered to assess a participant’s tendency to explore environments. The scale contains 8 items, with 4 positively stated and 4 negatively stated items. Responses are based on a 7-point scale, and are averaged (with negatively stated items being reverse scored). A higher score indicates that the participant is more likely to explore environments.

#### Measure of navigational growth mindset

The Navigation Growth Mindset scale (He & Hegarty, 2020), adapted from the General Growth Mindset scale (De Castella & Byrne, 2015), replaces the word “intelligence” in the original survey to “navigation ability” in the navigation version. Before completing the scale, participants are given a definition of “navigation ability,” and must indicate, out of several options, which ones demonstrate navigation ability (to ensure that participants have the same conceptual definition of what constitutes navigation ability before answering the items). Questions are on a 7-point scale, where a higher score indicates more growth mindset, i.e., belief that someone can improve their navigation ability. Items range from 1/strongly agree to 7/strongly disagree, items that indicate more navigational growth mindset (e.g., “I can always substantially change how good I am at navigating”) are reverse-scored.

#### Measure of social desirability

The Social Desirability Scale (Vésteinsdóttir et al., 2017) assesses the bias to answer the socially desirable response (i.e. the response viewed favorably by others) on self-report measures. The scale contains 10 true or false statements that a participant must indicate whether or not it applies to them. If they indicate “True” for the statement “I’m always willing to admit when I make a mistake” (the more favorable response), then that item is coded as a 1. If they indicate “False” to that same question, the item is coded as a 0. The total score ranges from 0 (no bias) to 10 (extreme bias).

#### Measure of mental rotation

The mental rotation test (MRT) (Vandenberg & Kuse, 1978) was administered online as a test of small-scale spatial ability. There are a total of 20 items in the task, broken up into 2 sections with 10 items and a time limit of 3 min per section. Participants are shown a reference image of an object made up of three-dimensional cubes. Participants must identify, out of 4 possible answers, which 2 are the same object, but rotated at a different angle. There are always 2 correct and 2 incorrect responses, and participants must get both of the responses correct to get a score of 1—otherwise, a score of 0 is received (this includes any items that they do not complete due to being timed out). The score is the sum of scores for the 20 items (possible range of 0 to 20).

Additional measures of ability and personality were included in connection with another study, and will be reported in a separate publication. These measures included the Cognitive Reflection Test-2 (Thomson & Oppenheimer, 2016), Need for Cognition Scale (Cacioppo et al., 1984), Brief Self-Control Scale (Maloney et al., 2012), Mindful Attention Awareness Scale (Brown & Ryan, 2003), General Growth Mindset Scale (De Castella & Byrne, 2015), Wordsum Plus (Cor et al., 2012), International Personality Item Pool-Big Five Markers-20 Item Questionnaire (Topolewska et al., 2014), and the Interest & Deprivation Epistemic Curiosity Scale (Litman & Spielberger, 2003).^2^

### Procedure

Immediately following sign-up, participants were given a Qualtrics link to the study. After providing consent, they completed all of the measures in a random order, except that the GPS Dependency Scale was always shown before the GPS Usage Scale. Following completion of these measures, participants were given an attention check, in which they were asked to recall a previous response within the Spatial Anxiety scale’s (He & Hegarty, 2020) questions, as well as a Captcha check (a measure used to identify and remove bot responses, in which a participant must click a box with their mouse stating they are a human) and a question indicating whether or not they took the survey seriously. Finally, participants responded to basic demographics (age, sex, ethnicity, and native language).

## Method (in-person study)

### Participants

A total of 67 participants were recruited to participate in the in-person study. This sample size was determined by an a priori power analysis, which revealed that 59 participants was minimum sample size necessary to achieve 80% power for detecting a medium effect (*r* = 0.35, as found in Ruginski et al., 2019), at a significance criterion of Alpha = .05. In this study, 6 participants were removed due to indicating they did not take the study seriously (2), or not completing all the measures due to participant time constraints or technical issues (4). Data from 61 participants were analyzed. Participants ranged from 18 to 32 years old (*M* = 19.67, SD = 2.63, 30 females). Participant ethnicities were White (*n* = 24, 39.3%), Asian (*n* = 15, 24.6%), Hispanic or Latino (*n* = 15, 24.6%), Multiracial (*n* = 2, 3.3%), Native Hawaiian or Pacific Islander (*n* = 1 1.6%), or Other (*n* = 4, 6.6%).

### Materials

For the in-person study, participants were administered all measures included in the online study, with the exception of the MRT (Vandenberg & Kuse, 1978) and the Social Desirability Scale (Vésteinsdóttir et al., 2017). The following measures were also administered, in addition to the Mindful Attention Awareness Scale (not reported—Brown & Ryan, 2003).^3^

#### Measure of large-scale navigation ability

The Virtual SILCton task (Weisberg et al., 2014) is an assessment of navigation ability administered via a desktop computer, keyboard, and mouse. In this task, participants first memorize the names and locations of 8 different buildings in a university campus-like environment, while following a series of guided routes. The first 2 routes are completely separate from each other, and include 8 buildings (landmarks) to be learned, and the remaining routes connect the first 2 to allow for an integrated representation of the environment. The learning phase is not timed, and participants traverse each route once in both the forward and backward direction.

In the onsite pointing task, participants are teleported to a building previously learned within the environment (facing the building). From there, they use their mouse to rotate their viewing direction on a horizontal plane to position a crosshair and indicate the locations of each of the other buildings learned, with the target building indicated on top of the screen on each trial. When the participant has determined the location of a building (assuming a straight line between the starting and target building), they click the left button on the mouse to record the projected angular position of the target. The absolute angular error between the building’s actual direction and the participant’s estimated direction is measured for each trial and averaged for within-route trials (buildings along the same initial learning routes) and between-route trials (buildings that were along different initial learning routes) trials separately. A higher score for the pointing task indicates more error, i.e., poorer performance. Each participant completes 24 within-route trials and 32 between-route trials.

In the map reconstruction task, participants are given a blank map and instructed to use their mouse to drag-and-drop the 8 buildings previously learned into their correct places within the environment, assuming an aerial perspective. Participants are not timed. An *R*^2^ value is calculated through a bidimensional regression (Friedman & Kohler, 2003) to determine the similarity (or shared variance) between the participant’s perceived *XY* coordinates of the 8 buildings in the environment and their actual *XY* coordinates. A higher *R*^2^ indicates more shared variance between the maps, meaning better performance.

All the tasks measured in the Virtual SILCton environment were aggregated into one “navigation” score for analyses, by transforming the absolute angular errors (reverse-scored so higher values indicate better performance) and *r*-squared values into *z*-scores, then averaging across the 3 values.

#### Measure of perspective-taking ability

The computerized Spatial Orientation Test (SOT, Friedman et al., 2020) is an assessment of spatial perspective taking ability, administered via a desktop computer, keyboard, and mouse. Participants are shown a 2D array of objects on a screen and asked to imagine they are standing on one object, facing another object, and asked to point to where a third object would be from this perspective (indicated by rotating a line on an arrow circle). There are 12 trials in this task, and participants are given 5 min to complete all of them. The absolute angular error between the third object’s actual position and the participant’s estimated position of the object are calculated and then averaged into one score, with timed-out responses classified as having 90 degrees of error (chance level performance). A higher score indicates poorer performance.

### Procedure

Participants came to the laboratory one at a time to participate in a small lab room. After providing consent, they were directed to the Virtual SILCton task (Weisberg et al., 2014) and completed the learning phase, the onsite pointing task, and finally the map reconstruction task. Participants were then administered the computerized SOT (Friedman et al., 2020), and afterwards were directed to complete the self-report measures. The questionnaires were administered in a random order except for the GPS scales being grouped together, as in the online study. Finally, participants completed demographics questions (age, sex, and ethnicity), an attention check, a Captcha, and a seriousness check.

## Analysis plan

We excluded participants who: (1) were flagged for straight-lined responses (i.e., they gave the same response to a series of questions either in one or more questionnaires), (2) indicated at the end of the survey that they did not take the study seriously, (3) failed the attention check at the end, that is, were completely incorrect when recalling their previous response to a question in the Spatial Anxiety Scale, (4) were identified as extreme outliers on a measure (> 3 SDs), and 5) did not complete the study in its entirety.

Analyses included: (1) Independent samples *t*-tests to examine whether there were any differences in responses to the questionnaires across the online and in-person studies the, (2) correlations (Pearson’s *r*) among all the variables of interest, with Bonferroni correction, (3) examination of the differences between correlations among SBSOD, spatial anxiety and each reported GPS use, based on Zou’s confidence interval (2007) in the “cocor” package in R (Diedenhofen & Musch, 2015), (4) a one-way ANOVA to analyze differences in the frequency of using each GPS functions (turn-by-turn directions, route planning, time and traffic estimates, and finding a specific service along a route), and finally (5) mixed ANOVAs to examine whether GPS use was moderated by self-reported sense of direction (high vs low, based on a median split) and familiarity of the navigation scenario (never been before, traveled 1–5 times, traveled 5–10 times, and traveled more than 10 times, followed by post-hoc tests. Analysis code, the data, and more in-depth discussion on each variable’s coding scheme can be found at https://osf.io/yn4jr/ (for the online study) and https://osf.io/bzcrp/ (for the in-person study).

## Results

### Comparison of scores in the online and in-person studies

Welch’s independent samples *t*-tests were conducted to test for significant mean differences between the self-report measures in the online and in-person studies. None of these measures were significantly different between studies; reported GPS dependency, *t*(103.29) = 0.25, *p* = 0.801, GPS use for turn-by-turn directions, *t*(116.14) = − 0.27, *p* = 0.785, GPS use for route planning, *t*(110.86) = − 0.75, *p* = 0.454, GPS use for time and traffic estimates, *t*(106.94) = 0.12, *p* = 0.908, GPS use for finding a specific service along a route, *t*(110.40) = 0.21, *p* = 0.834, sense of direction, *t*(95.12) = 1.18, *p* = 0.242, spatial anxiety, *t*(100.30) = − 0.32, *p* = 0.750, navigational growth mindset, *t*(92.73) = − 0.36, *p* = 0.721, or exploration tendency, *t*(92.16) = 0.03, *p* = 0.980. Therefore, we collapsed results from the two studies when analyzing these measures, for a final sample size of 249 participants in the aggregated analyses.

Descriptive statistics, reliability coefficients (Omega), and measures of skewness and kurtosis are reported in Table 1. Omegas ranged from 0.70 to 0.94, indicating adequate to high reliability for all the measures and none of the measures departed from normality.

**Table 1:** Descriptive statistics for the measures in the online (188) and in-person (61) studies

Variable	*N*	Omega	*M*	SD	Skewness	Kurtosis
1. SOD	249	0.90	3.90	1.01	0.03	− 0.22
2. Spatial anxiety	249	0.93	2.76	0.86	0.23	− 0.62
3. Exploration tendency	249	0.89	3.74	1.09	− 0.12	− 0.38
4. Navigational growth mindset	249	0.94	4.61	1.10	− 0.30	− 0.47
5. GPS dependency	249	0.91	3.56	0.83	0.09	− 0.89
6. Turn-by-turn GPS	249	0.87	0.96	0.41	0.28	0.09
7. Route planning GPS	249	0.92	0.90	0.52	0.06	− 0.68
8. Time and traffic estimates GPS	249	0.91	1.21	0.48	− 0.24	− 0.40
9. Finding a specific service GPS	248	0.91	0.89	0.47	0.10	− 0.48
10. Social desirability	188	0.70	3.94	2.27	0.21	− 0.54
11. Mental rotation	188	0.85	7.20	4.62	0.38	− 0.58
12. Spatial orientation test^†^	61	−	3.33	0.89	− 0.42	− 0.81
13. Onsite pointing (within-route)	61	0.94	23.16	16.36	0.56	− 1.19
14. Onsite pointing (between-route)	61	0.91	50.29	20.71	− 0.22	− 0.80
15. Map reconstruction	61	–	0.65	0.27	− 0.59	− 0.95

^†^The spatial orientation test was log-transformed to satisfy the assumption of normality

*Skewness:* all measures had a value between − 0.59 and 0.56, and were within a normal range of values. *Kurtosis:* all measures had a value between − 1.19 and 0.09, and were within a normal range of values

#### **Research Question 1**

How are self-reports of navigation ability (sense of direction) and spatial anxiety related to GPS use and dependency?

Pearson correlations between the measures are reported in Table 2. Bonferroni correction for multiple comparisons indicated an adjusted Alpha = 0.001, which was used to interpret the significance of correlations of the self-report and online study variables. Significant differences in correlations are reported using Zou’s confidence interval (2007). For differences in correlations, Bonferroni correction for multiple comparisons indicated an adjusted Alpha = 0.01. With respect to reported GPS use, using GPS for turn-by-turn directions was significantly negatively correlated with SOD, 99.9% CI [− 0.61, − 0.28], and positively correlated with spatial anxiety, 99.9% CI [0.14, 0.51], as predicted. Reported use of GPS for finding a specific service was also negatively correlated with SOD, 99.9% CI [− 0.46, − 0.08], and positively correlated with spatial anxiety, 99.9% CI [0.06, 0.44]. However, reported use of GPS for time and/or traffic estimates was not significantly correlated with SOD, 99.9% CI [− 0.37, 0.03], or spatial anxiety, 99.9% CI [− 0.03, 0.37]. Reported use of GPS for route planning was significantly positively correlated with spatial anxiety, 99.9% CI [0.06, 0.45], but not with SOD, 99.9% CI [− 0.38, 0.01]. In summary, people with a poor sense of direction reported using GPS more frequently for turn-by-turn directions and finding a specific service along a route. Additionally, people with higher spatial anxiety reported using GPS more frequently for all GPS uses except time and traffic estimates. However, reported use of GPS for turn-by-turn directions was significantly more correlated with SOD than route planning, 99% CI for the difference [− 0.33, − 0.11], time and traffic estimates, 99% CI [− 0.44, − 0.13], and for finding a specific service, 99% CI [− 0.34, − 0.01]. Reported use of GPS for turn-by-turn directions was significantly more correlated with spatial anxiety than time and traffic estimates, 99% CI [0.003, 0.31], but no other correlations between reported GPS use and spatial anxiety were significantly different from each other.

**Table 2:** Correlations between the self-report variables (combined online and in-person studies)

Variable	1	2	3	4	5	6	7	8	9
l. SOD	–								
2. Spatial anxiety	− .52*	–							
3. Exploration tendency	.50*	− .40*	–						
4. Navigational growth mindset	.34*	− .32*	.19	–					
5. GPS dependency	− .56*	.43*	− .28*	− .29*	–				
6. Turn-by-turn GPS	− .46*	.34*	− .20	− .22*	.55*	–			
7. Route planning GPS	− .19	.27*	.01	− .14	.22*	.44*	–		
8. Time and traffic estimates GPS	− .18	.18	− .02	− .18	.29*	.50*	.53*	–	
9. Finding a specific service GPS	− .28*	.26*	− .11	− .20	.35*	.39*	.44*	.50*	–
10. Social desirability	.21	− .05	− .04	.15	− .09	− .14	− .02	− .15	− .03

*p < .001

Correlations that survived the Bonferroni correction for multiple comparisons (Bonferroni corrected alpha =.001) are inidicated by correlations that contains 1 star (i.e., *p* < .001)

General GPS dependency had a large positive correlation with using GPS for turn-by-turn directions, 99.9% CI [0.39, 0.68]. While GPS dependency was also significantly positively correlated with reported use of the other GPS functions (see Table 2), turn-by-turn directions had a significantly higher correlation with GPS dependency than route planning, 99% CI [0.18, 0.49], time and traffic estimates, 99% CI [0.12, 0.40], and for finding a specific service, 99% CI [0.05, 0.36]. No other correlations between GPS dependency and reported GPS use differed significantly. In sum, and as predicted, participants who depend more on GPS are particularly likely to report using GPS for turn-by-turn directions while they are navigating.

#### **Research Question 2**

How do people use GPS and moderate their use of GPS based on familiarity of the navigation scenario?

#### Frequencies of different GPS uses

**Fig. 1:**
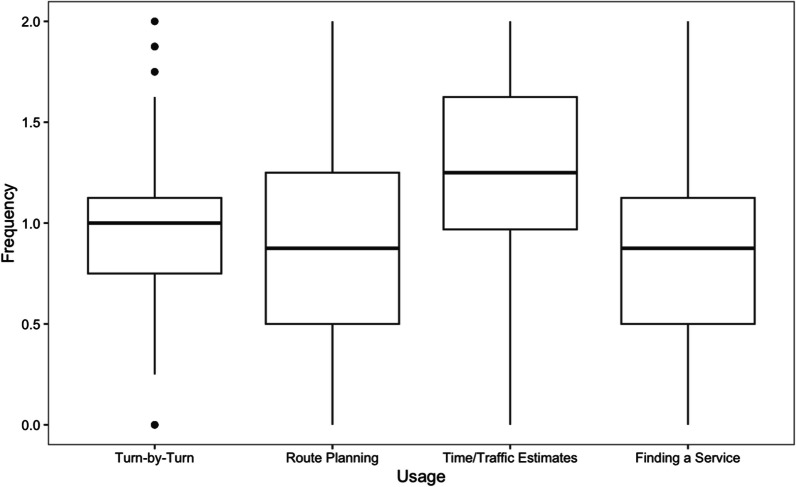
Frequency boxplots for each function of GPS across navigation scenarios (combined online and in-person studies)

Frequencies of reported uses of four GPS functions (turn-by-turn directions, route planning, time and traffic estimates, and finding a specific service) are shown in Fig. 1. A one-way repeated measures ANOVA comparing the reported frequency of using these four functions indicated a significant effect of GPS function, *F*(3,741) = 47.33, *p* < 0.001, ${\eta }^{2}$ = 0.07. Post-hoc analyses using Tukey’s HSD indicated that the average reported frequency of using GPS for time and traffic estimates (*M* = 1.21) was significantly higher than reported use of GPS for turn-by-turn directions (*M* = 0.96), route planning (*M* = 0.90), and finding a specific service (*M* = 0.89), *p* < 0.001 in all these comparisons. There were no significant differences in reported frequencies of GPS use for other functions (i.e., besides time and traffic estimates). In sum, people reported to using GPS most frequently for time and traffic estimates, and more often than any other GPS function*.*

#### Effects of environment familiarity

**Fig. 2:**
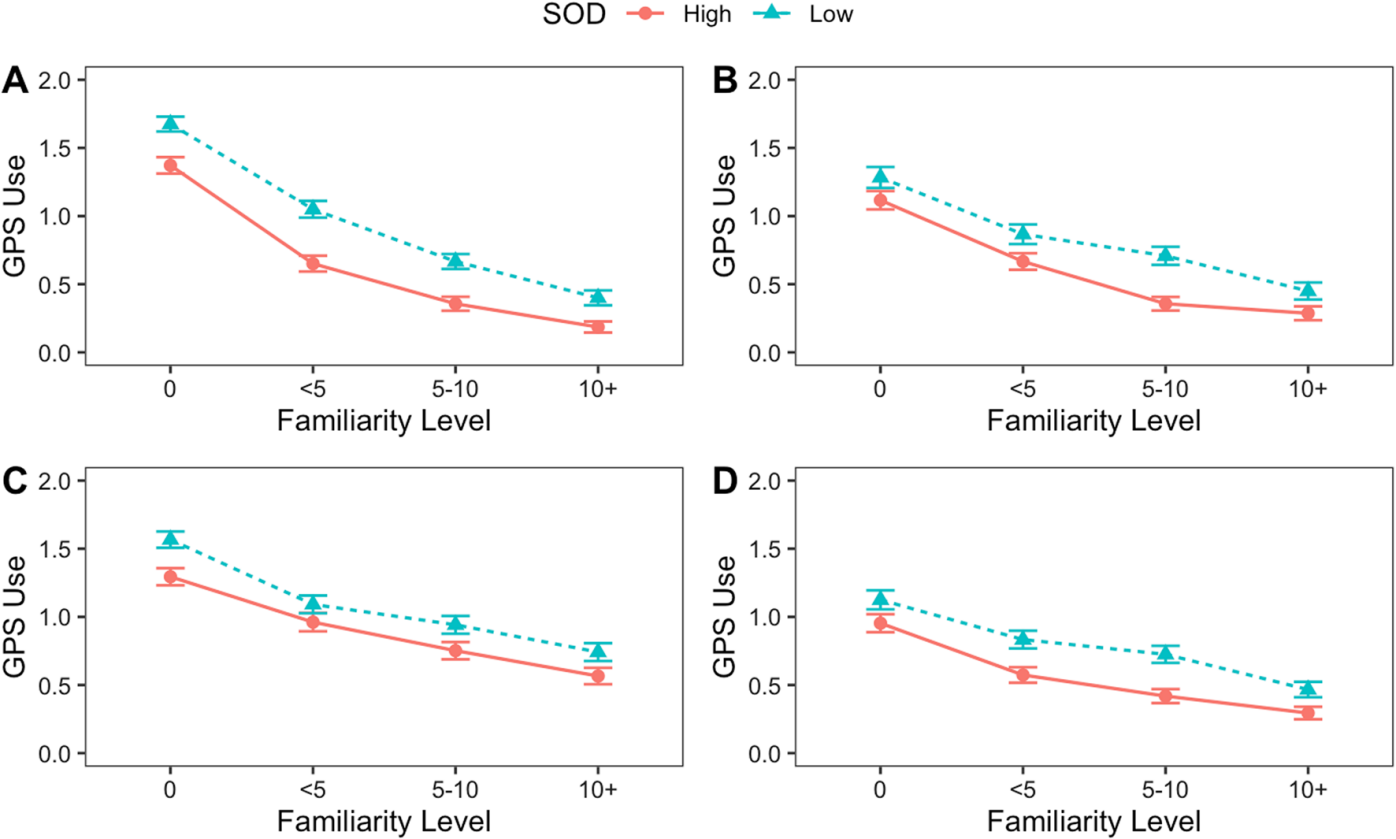
Interaction plots for reported GPS functions across scenario familiarity with SOD (combined online and in-person studies).** A**: Turn-by-turn direction GPS function; **B**: route planning GPS function; **C**: time and traffic estimate GPS function; **D**: finding a service GPS function

To assess the effect of navigation scenario familiarity, a 4 × 2 ANOVA was conducted on the reported frequency of using each GPS functions across 4 levels of familiarity (never traveled, traveled less than 5 times, traveled 5–10 times, and traveled more than 10 times) and high vs. low SOD, based on a median split (see Fig. 2). For turn-by- turn directions, there was a significant main effect of SOD, *F*(1,988) = 62.69, *p* < 0.001, partial ${\eta }^{2}$= 0.06, and a significant main effect of familiarity,* F*(3,988) = 192.59, *p* < 0.001, partial ${\eta }^{2}$ = 0.37, but no significant interaction, *F*(3,988) = 0.95, *p* = 0.415, ${\eta }^{2}$ = 0.003. High SOD people (*M* = 0.64) reported using GPS less for turn-by-turn directions than low SOD people (*M* = 0.95). Reported use of GPS for turn-by-turn directions decreased as the familiarity of a navigation scenario increased (see Fig. 2a). Post hoc (Tukey’s HSD) tests indicated that all familiarity levels were significantly different from each other (*p* < 0.001 for all comparisons).

For route planning, there was a significant main effect of SOD, *F*(1,988) = 24.05, *p* < 0.001, partial ${\eta }^{2}$ = 0.02, and a significant main effect of familiarity, *F*(3,988) = 64.77, *p* < 0.001, partial ${\eta }^{2}$ = 0.16, but no significant interaction, *F*(3,988) = 0.98, *p* = 0.401, partial ${\eta }^{2}$ = 0.003. High SOD people (*M* = 0.61) reported using GPS less for route planning than low SOD people (*M* = 0.83). Reported use of GPS for route planning decreased as the familiarity of a navigation scenario increased (see Fig. 2b). Post-hoc (Tukey’s HSD) tests indicated that with the exception of scenarios traveled 5–10 times before compared to more than 10 times (*p* = 0.056), the remaining comparisons were significantly different, *p* < 0.01 in all cases.

For time and traffic estimates, there was a significant main effect of SOD, *F*(1,988) = 18.17, *p* < 0.001, partial ${\eta }^{2}$ = 0.02, as well as a significant main effect of familiarity, *F*(3,988) = 53.90, *p* < 0.001, partial ${\eta }^{2}$ = 0.14, but no significant interaction, *F*(3,988) = 0.43, *p* = 0.731, partial ${\eta }^{2}$ = 0.001. High SOD people (*M* = 0.89) reported using GPS less for time and traffic estimates than low SOD people (*M* = 1.09). Reported use of GPS for time and traffic estimates decreased with familiarity of the navigation scenario (see Fig. 2c). Post-hoc (Tukey HSD) tests indicated that all differences between levels of familiarity were significant (*p* < 0.05 in all comparisons).

For finding a service, there was a significant main effect of SOD, *F*(1,988) = 29.21, *p* < 0.001, partial ${\eta }^{2}$ = 0.03, and a significant main effect of familiarity, *F*(3,988) = 43.58,* p* < 0.001, partial ${\eta }^{2}$ = 0.12, but no significant interaction, *F*(3,988) = 0.63, *p* = 0.593, partial ${\eta }^{2}$ = 0.002 High SOD people (*M* = 0.56) reported overall using GPS less for finding a specific service than low SOD people (*M* = 0.79). Reported use of GPS for finding a specific service decreased as the familiarity of a navigation scenario increased (see Fig. 2d). Post-hoc (Tukey HSD) tests indicated that with the exception having traveled 1–5 times before and traveled 5–10 times before (*p* = 0.116), the remaining comparisons were significantly different (*p* < 0.01 in these cases).

It is possible that participants reported less frequent use of GPS in more familiar scenarios because they perceived that to be the socially desirable response (i.e., how much people *believe* they should be using and depending on GPS, rather than actual behaviors). However, none of the correlations between GPS use and dependency were significantly correlated with social desirability (see Table 2), suggesting that this was not a major influence on participants’ responses to the questionnaires. While there are overall trends that high and low SOD converge as familiarity increases, the interaction was not significant for any GPS function. In sum, as the familiarity level of a navigation scenario increased, reported usage of GPS for all functions subsequently decreased, and those with high SOD reported using every GPS function less than those with low SOD. However, all participants modulated their reported use of GPS depending on their familiarity with the environment, regardless of the GPS function and their SOD.

##### **Research Question 3**

How is objectively measured navigation ability related to GPS dependency and use?

In the in-person study, all measures in the Virtual SILCton task (Weisberg et al., 2014) were significantly correlated with each other: within-route and between-route pointing, *r*(59) = 0.50, *p* < 0.001, 95% CI [0.29, 0.67], within-route pointing and map reconstruction, *r*(59) = 0.65, *p* < 0.001, 95% CI [0.48, 0.78], and between-route pointing and map reconstruction, *r*(59) = 0.64, *p* < 0.001, 95% CI [0.47, 0.77] justifying their combination into a single measure of navigation performance, consistent with previous research on this task (Weisberg et al., 2014).^4^

We predicted that people who reported greater general dependency on GPS and more use of GPS for turn-by-turn directions would have poorer objective navigation performance, as measured by Virtual SILCton, and explored the relation between objective navigation performance and the other GPS uses. Bonferroni correction indicated an adjusted Alpha = 0.01, which was used to interpret the significance of correlations between navigation performance and the GPS measures. Objective navigation performance (as measured by SILCton) significantly negatively correlated with GPS dependency, *r*(59) = − 0.34, *p* = 0.008, 99% CI [− 0.60, − 0.01]. However, none of the correlations between objective navigation performance and reported use of GPS were significant; for turn-by-turn directions, *r*(59) = − 0.23, *p* = 0.071, 99% CI [− 0.52, 0.10], route planning, *r*(59) = 0.01, *p* = 0.951, 99% CI [− 0.32, 0.33], time and traffic estimates, *r*(59) = − 0.002, *p* = 0.991, 99% CI [− 0.33, 0.32], or finding a specific service, *r*(59) = − 0.23, *p* = 0.077, 99% CI [− 0.52, 0.11].

In summary, better performance on the Virtual SILCton tasks was associated with lower overall GPS dependency. While in the predicted direction, correlations with reported use of specific functions of GPS (turn-by-turn navigation and finding a specific service) and navigation performance did not reach statistical significance.

### Replication of He and Hegarty (2020) and Ruginski et al. (2019)

We examined whether the self-report measures replicated the findings of He and Hegarty (2020), specifically, correlations between GPS dependency, spatial anxiety, exploration tendency, and navigational growth mindset. After correcting for multiple comparisons (Alpha = 0.001) in Study 1, significant correlations were found between GPS dependency and SOD, *r*(247) = − 0.56, *p* < 0.001, 99.9% CI [− 0.69, − 0.40], spatial anxiety, *r*(247) = 0.43, *p* < 0.001, 99.9% CI [0.25, 0.59], exploration tendency, *r*(247) = − 0.28, *p* < 0.001, 99.9% CI [− 0.46, − 0.08], and navigational growth mindset, *r*(247) = − 0.29, *p* < 0.001, 99.9% CI [− 0.47, − 0.09]. These findings replicate those of He & Hegarty. The only finding that did not survive multiple comparisons corrections was the association between exploration tendency and navigational growth mindset, *r*(247) = 0.19, *p* = 0.003, 99.9% CI [− 0.02, 0.38].

We also examined whether GPS dependency was related to mental rotation or perspective taking as small-scale measures of spatial ability were mediators of the relationship between GPS dependency in the study by Ruginski et al. (2019). Note that mental rotation was measured in the online study alone and perspective taking was measured in the in-person study alone. GPS dependency was not correlated with mental rotation ability in the online study, *r*(186) = − 0.12, *p* = 0.116 or with perspective taking in the in-person study *r*(59) = 0.18, *p* = 0.178. In sum, GPS dependency was not significantly associated with mental rotation or perspective taking. Therefore, we failed to replicate Ruginski et al. (2019) in regards to GPS dependency and its association with small-scale measures.

## Discussion

The goals of the present research were to examine how reported use of specific GPS functions varies across individuals and situations, and how reported GPS use relates to measures of navigation ability. Specifically, it addressed the following questions: (1) How are self-reports of navigation ability (sense of direction) and spatial anxiety related to GPS use and dependency? (2) How do people use GPS and moderate their use of GPS based on familiarity of the navigation scenario? and (3) How is objectively measured navigation ability related to GPS dependency and use?

How are self-reports of navigation ability (sense of direction) and spatial anxiety related to GPS use and dependency?

This study provides new information about how people report using GPS navigation devices, and how that differs across self-reported spatial abilities. First, those who report being more dependent on using GPS for navigating also report using all GPS functions more frequently, but the association between GPS dependence and reported GPS use is strongest for turn-by-turn directions. Second, higher self-reported sense of direction (SOD) is correlated with less reported use of GPS for turn-by-turn directions and finding a specific service along a route, but is not correlated with reported use of GPS for time and traffic estimates, or route planning. The association between SOD and reported use of GPS was also greatest for the reported use of GPS for turn-by-turn directions.

Third, spatial anxiety is correlated with more reported use of GPS for all functions except time and traffic estimates. It is possible that using GPS for time and traffic estimates is not related to perceived spatial ability or anxiety as this function of GPS is supplemental to knowledge of the layout of an environment (i.e. a cognitive map). A cognitive map is generally thought of as a static spatial representation of an environment, whereas time and traffic estimates augment this spatial representation with dynamic information about current conditions, such as traffic. The results support the idea that low SOD and spatially anxious people use GPS because they are not confident in their own ability to form cognitive maps, so they use GPS to replace internal spatial knowledge. In contrast, as the use of GPS for time and traffic estimates is independent of knowledge of the layout of an environment (i.e. a cognitive map) it is less related to self-reported sense of direction and spatial anxiety.

How do people use GPS and moderate their use of GPS based on familiarity of the navigation scenario?

In these studies, people reported that they modulate their use of GPS depending on their familiarity with a navigation scenario. Apparently, even those who are highly dependent on GPS use navigation devices less in more familiar environments and navigation tasks (or at least believe they do). Rather than painting a picture of people blindly relying on their GPS devices and ignoring their environmental knowledge (McKinlay, 2016), this is the first result, to our knowledge, indicating that people most commonly report using these devices to supplement their spatial knowledge. First, they report using GPS more when they have less knowledge or familiarity with an area. Second, across all navigation scenarios, the most common reported use of GPS is for time and traffic estimates, that is, the function that contains supplemental information to a cognitive map. While the reported use of this GPS function decreases as navigation scenarios become more familiar, it is possible that this is attributable to more familiarity with the probability of specific traffic patterns in more familiar environments. Finally, it is also observed that people with a good sense of direction generally report using GPS less than those with poor SOD, possibly because they are more able to construct accurate spatial representations of environments and therefore need to use GPS less in general.

How is objectively measured navigation ability related to GPS dependency and use?

The in-person study replicated the result that people with greater dependence on GPS for navigating have poorer navigation performance when tasked with learning the layout of a novel desktop environment (in Virtual SILCton), replicating Ruginski et al. (2019) but with a more robust measure of GPS dependence (8 questions, compared to 1). This is an important finding adding to the evidence that GPS dependence is negatively related to objective measures and not just self-reported measures of navigation. However, Virtual SILCton performance was not significantly associated with reports of any specific GPS function (turn-by-turn directions, route planning, time and traffic estimates, or finding a specific service).

### Limitations and future directions

A limitation of this study is that it is correlational so any associations among reported GPS use, spatial anxiety and navigation ability in these studies cannot indicate direction of causality. In other words, we cannot determine whether those with poor sense of direction and high spatial anxiety use GPS more because they are not confident in their navigation abilities, whether more use of GPS impairs the development of navigation ability, or if there is a cyclic relationship among the variables. Likewise, we cannot make any causal statements about the relation between navigation ability and GPS dependence with the data presented here.

Another possible limitation is that self-reports of using GPS may not be entirely accurate, as people may respond to how much they *believe* they should use each function, rather than how much they actually use it in specific navigation scenarios. However, this limitation is somewhat alleviated by the fact that there were no significant correlations between measured social desirability bias and GPS uses (see Table 2).

The measure for reported GPS use was on a 3-point scale, and therefore may not be sensitive enough to pick up gradations of each function and maintain consistency across studies (Lozano et al., 2008). This can be addressed in a future study by actually tracking how much people use their GPS, and in what ways they use it, rather than relying on self-reports which rely on a person’s memory of their usage habits. Additionally, it is possible that the measure does not cover all of the novel ways GPS can be used, although it is noted that the scale was based on open ended reports of how people use GPS in our pilot study.

Examining relations between reported GPS use and objectively measured navigation ability was limited by using an environment learned entirely via a desktop computer (for example, because there was no physical locomotion occurring when learning or completing tasks in the environment). Navigating in real environments includes spatial updating-based cues (proprioception, vestibular signals, and motion efference copy) in addition to the visual cues afforded in desktop virtual environments and navigation ability in real environments is partially dissociated from navigation ability based on visual input alone (Hegarty et al., 2006). It is to be noted, however, that the Virtual SILCton task was based on real-world studies, such as Ishikawa & Montello (2006) and Schinazi et al. (2013), and has demonstrated itself as a reliable measure in previous work (Ruginski et al., 2019; Weisberg & Newcombe, 2016; Weisberg et al., 2014; Youngson et al., 2019). Additionally, this concern is alleviated by a recent study in which Zhou (2022) found evidence that GPS dependency and reported GPS use were related to objective measures of navigation—wayfinding efficiency, taking shortcuts, and pointing ability—in a real-world environment.

A future study could involve a longitudinal design to track GPS use for an extended period of time and observe its effects on navigation ability to infer direction of causality (similar to Dahmani & Bohbot, 2020). It is possible that the age of adoption of GPS matters, such that adopting GPS in earlier, more formative years (e.g., at or before one learns to drive) has more detrimental effects than adopting GPS later in life.

Finally, modifications can be made to the GPS scales to better parse out reported frequencies of using various components of GPS. Although the scales had good reliability (see Table 1), some items on these scales had low variability (see Additional file 1: Tables S1 and S2), suggesting that some revision of the scales is warranted to improve their scale psychometric properties. This could potentially be achieved in a few ways: (1) revising the scale to a 5-point scale to better distinguish gradations of use, (2) replacing the turn-by-turn direction function by a more dynamic scale, where participants can indicate how much of their route requires turn-by-turn directions from the GPS, and (3) including navigation scenarios with substantial response variability.^5^ This should help to better discriminate reported GPS use, as well as provide more insights as to exactly how much someone needs to use turn-by-turn directions.

## Conclusion

In conclusion, this study has taken a novel approach to examine how GPS technologies are used and how specific uses are related to general spatial ability (both perceived and objective) and perceived dependence on GPS. Through a robust objective measure of navigation ability, we can better conclude that reported dependency on GPS is associated with poorer spatial knowledge acquisition. Additionally, people most strongly associate their perceived dependence on GPS with the frequency at which they report using GPS for turn-by-turn directions. In light of these associations, we also found that GPS is reported as being generally used most for time and traffic estimates when navigating, and people report modulating their overall use as navigation scenarios become more familiar. In other words, people report using GPS to some extent intelligently, generally using it to augment navigation ability. Delineating among the specific functions of GPS and their associations with spatial cognition will provide a strong basis for the future modifications and improvements of GPS for newer generations.

### Supplementary Information

**Additional file 1.** Individual item descriptive statistics for the GPS scales used, and correlations for each of the measures in the Virtual SILCton (in-person) study.

## Data Availability

The datasets generated and analyzed during the current studies are available in the OSF repository, for both the online study (https://osf.io/yn4jr/) and the in-person study (https://osf.io/bzcrp/).

## Appendix

### GPS usage questionnaire

Please consider the following navigation scenarios. For each scenario, you will be given a list of different ways GPS can be used to navigate an area (descriptions below). Please mark how often you use **each function** for **each scenario** (Never, Sometimes, Often/Always).

Different ways of using GPS:

- *Turn-by-turn navigation*: Following the GPS' instructions for the **entire** route (for example, every turn given).
- *Route planning*: Using GPS to plan your route, at **home**, before you start your journey.
- *Time/traffic estimates*: Using GPS to know how long it will take to reach your destination (either for your own purposes or someone else’s, e.g. sending a pin), and/or to know if traffic/congestion is going to affect your route.
- *Finding a specific service*: You are using the GPS to find a service, such as the nearest gas station, convenience store, coffee shop, etc. on your way to your destination.

1. Navigating from your current residence to school and/or work, assuming the place(s) is/are** 0–15 min** away.

**Table:**

	Never	Sometimes	Often/always
Turn-by-turn navigation	0	1	2
Route planning	0	1	2
Time/traffic estimates	0	1	2
Finding a specific service (e.g., a gas station) along the way	0	1	2

2. Navigating from your current residence to school and/or work, assuming the place(s) is/are** more than 15 min** away.

**Table:**

	Never	Sometimes	Often/always
Turn-by-turn navigation	0	1	2
Route planning	0	1	2
Time/traffic estimates	0	1	2
Finding a specific service (e.g., a gas station) along the way	0	1	2

3. Navigating from your current residence to a place in or near your hometown (e.g., a mall, salon, trail, etc.), assuming you have** never** traveled to this place before.

**Table:**

	Never	Sometimes	Often/always
Turn-by-turn navigation	0	1	2
Route planning	0	1	2
Time/traffic estimates	0	1	2
Finding a specific service (e.g., a gas station) along the way	0	1	2

4. Navigating from your current residence to a place in or near your hometown (e.g., a mall, salon, trail, etc.) that you have been to** no more than 5 times** before.

**Table:**

	Never	Sometimes	Often/always
Turn-by-turn navigation	0	1	2
Route planning	0	1	2
Time/traffic estimates	0	1	2
Finding a specific service (e.g., a gas station) along the way	0	1	2

5. Navigating from your current residence to a place in or near your hometown (e.g., a mall, salon, trail, etc.) that you have been to** only 5–10 times** before.

**Table:**

	Never	Sometimes	Often/always
Turn-by-turn navigation	0	1	2
Route planning	0	1	2
Time/traffic estimates	0	1	2
Finding a specific service (e.g., a gas station) along the way	0	1	2

6. Navigating from your current residence to a place in or near your hometown (e.g., a mall, salon, trail, etc.) that you have been to** more than 10 times before**.

**Table:**

	Never	Sometimes	Often/always
Turn-by-turn navigation	0	1	2
Route planning	0	1	2
Time/traffic estimates	0	1	2
Finding a specific service (e.g., a gas station) along the way	0	1	2

7. Navigating from your current residence to visit the home of a friend or family member in another city or town (that you visit** no more than a couple of times a year**), assuming the city is** at least 3 h away** from you.

**Table:**

	Never	Sometimes	Often/always
Turn-by-turn navigation	0	1	2
Route planning	0	1	2
Time/traffic estimates	0	1	2
Finding a specific service (e.g., a gas station) along the way	0	1	2

8. Navigating from your current residence to a hotel/Airbnb in a city or town that you have** never visited** before, assuming this city is** at least 3 h away** from you.

**Table:**

	Never	Sometimes	Often/always
Turn-by-turn navigation	0	1	2
Route planning	0	1	2
Time/traffic estimates	0	1	2
Finding a specific service (e.g., a gas station) along the way	0	1	2

## Abbreviations

GNSS: Global Navigation Satellite Systems
GPS: Global positioning system
LBS: Location based services
MRT: Mental rotation test
SBSOD: Santa Barbara Sense of Direction
SILCton: Spatial Intelligence Learning Center Test of Navigation
SOD: Sense of direction
SOT: Spatial orientation test

## Footnotes

1 In the online study, there were originally 5 GPS uses: turn-by-turn directions, minimal navigation (using turn-by-turn directions for only a small portion of the route), route planning, time and traffic estimates, and finding a specific service. The “minimal navigation” GPS use was removed for the in-person study as it was a confusing item for participants, and is consequently not included in analyses and their reports.

2 These measures were either not significant with the GPS measures, and/or correlations were too low to be considered in this manuscript (*r*-values less than 0.20).

3 The Mindful Attention Awareness Scale (Brown & Ryan, 2003) was not correlated with any of the GPS related measures, and therefore was not included in the report.

4 See Table S3 in Additional file 1 for a more comprehensive breakdown of the correlations between each Virtual SILCton measure and the remaining variables of interest. It is noted that the effects of Virtual SILCton measures are relatively similar to each other, justifying aggregating the 3 measures into one “Navigation” score.

5 It is noted that in the GPS Dependency and GPS Usage scales, some items had little variance in participant responses. We also conducted correlation analyses in the combined measures after removing items with little response variability (i.e., items 1 and 2 in the GPS Dependency scale, and items 2, 6, 7, and 8 in the GPS Usage scale), but results and findings did not change. As a result, we reported the full scales in this manuscript.
