# Supplementary material for: How is GPS used? Understanding navigation system use and its relation to spatial ability
Source: Cogn Res Princ Implic. 2024 Mar 19;9:16. doi: 10.1186/s41235-024-00545-x (PMC10951145; doi:10.1186/s41235-024-00545-x)
Supplement: Supplementary file 1 — Additional file 1. Individual item descriptive statistics for the GPS scales used, and correlations for each of the measures in the Virtual SILCton (in-person) study. [file 41235_2024_545_MOESM1_ESM.docx]

**Supplementary Materials**

**Table S1**

*Individual item descriptive statistics (N = 249) for the GPS Dependency Scale (He & Hegarty, 2020) for the combined online and in-person studies.*

| *Question/Item* | *M* | *SD* |
| --- | --- | --- |
| 1. Deciding which direction to walk in an unfamiliar city or town after coming out of a train/bus/metro station. | 4.34 | 0.82 |
| 2. Finding my way to an appointment in an unfamiliar area of a city or town. | 4.67 | 0.60 |
| 3. Leaving a store that I have been to a couple of times and deciding which way to turn to get to a destination. | 2.77 | 1.31 |
| 4. Finding my way back to a familiar area after realizing I have made a wrong turn. | 2.98 | 1.37 |
| 5. Returning from a familiar place to my home, if I have never gone home directly from this place before. | 2.88 | 1.36 |
| 6. Taking a novel detour to a familiar place, due to a roadblock on my usual route. | 3.11 | 1.36 |
| 7. Finding my way to an appointment in an area of a city or town with which I am only roughly familiar. | 3.74 | 1.12 |
| 8. Traveling to a new place after getting someone’s directions. | 4.01 | 1.05 |

*Note:* All items begin with the statement “Do you rely on GPS when:” before listing the scenario.

**Table S2**

*Individual item descriptive statistics (N = 249) for the GPS Usage Scale for the combined online and in-person studies.*

| *Question/Item* | *Turn-by-turn*  *directions*  *M (SD)* | *Route planning*  *M (SD)* | *Time & traffic estimates*  *M (SD)* | *Finding a specific service*  *M (SD)* |
| --- | --- | --- | --- | --- |
| 1. School and/or work, assuming the place(s) is/are 0-15 minutes away | 0.43 (0.68) | 0.53 (0.72) | 0.99 (0.78) | 0.62 (0.67) |
| 2. School and/or work, assuming the place(s) is/are more than 15 minutes away | 0.70 (0.76) | 0.78 (0.78) | 1.22 (0.74) | 0.76 (0.71) |
| 3. A place in or near your hometown (e.g. a mall, salon, trail, etc.), assuming you have never traveled to this place before | 1.52 (0.66) | 1.20 (0.81) | 1.43 (0.70) | 1.04 (0.76) |
| 4. A place in or near your hometown (e.g. a mall, salon, trail, etc.) that you have been to no more than 5  times | 0.84 (0.69) | 0.76 (0.74) | 1.02 (0.74) | 0.70 (0.69) |
| 5. A place in or near your hometown (e.g. a mall, salon, trail, etc.) that you have been to only 5-10 times before | 0.51 (0.61) | 0.53 (0.67) | 0.84 (0.72) | 0.57 (0.65) |
| 6. A place in or near your hometown (e.g. a mall, salon, trail, etc.) that you have been to more than  10 times before | 0.29 (0.54) | 0.37 (0.63) | 0.65 (0.70) | 0.38 (0.58) |
| 7. Visit the home of a friend or  family member in another city or town (that you visit no more  than a couple of times a year), assuming this city is at least 3 hours away from you. | 1.55 (0.65) | 1.42 (0.74) | 1.69 (0.57) | 1.37 (0.70) |
| 8. A hotel/Airbnb in a city or turn that you have never visited before, assuming this city is at least 3 hours away from you. | 1.84 (0.48) | 1.61 (0.66) | 1.82 (0.46) | 1.64 (0.61) |

*Note:* all items begin with the phrase “Navigating from your current residence to…”

Due to the “Minimal GPS” function being removed in most analyses and in the in-person study, it is not included in this table.

**Table S3**

*Correlations for each of the Measures in the Virtual SILCton Environment (In-Person Study)*

| *Variable* | Within-route Pointing | | Between-route Pointing | Map Reconstruction |
| --- | --- | --- | --- | --- |
| 1. SOT^†^ | | .12 | .15 | .15 |
| 2. SOD | | .23 | .15 | .18 |
| 3. Spatial Anxiety | | -.34* | -.32 | -.37* |
| 4. GPS Dependency | | -.35* | -.25 | -.27 |
| 5. Navigational Growth Mindset | | .10 | .18 | .09 |
| 6. Exploration Tendency | | .38* | .26 | .23 |
| 7. Turn-by-turn GPS | | -.18 | -.24 | -.18 |
| 8. Route Planning GPS | | .16 | -.09 | -.04 |
| 9. Time/Traffic Estimates GPS | | .10 | -.06 | -.05 |
| 10. Finding a Service GPS | | -.09 | -.23 | -.25 |

**p*<.01.

*Note:* Correlations that survived the Bonferroni correction for multiple comparisons (Bonferroni corrected alpha = .01) are indicated by correlations that contain 1 start (i.e., *p* < .01).

† SOT was log-transformed to satisfy the assumption of normality.
